# Structural Brain Alterations in Patients with Lumbar Disc Herniation: A Preliminary Study

**DOI:** 10.1371/journal.pone.0090816

**Published:** 2014-03-03

**Authors:** Michael Luchtmann, Yvonne Steinecke, Sebastian Baecke, Ralf Lützkendorf, Johannes Bernarding, Jana Kohl, Boris Jöllenbeck, Claus Tempelmann, Patrick Ragert, Raimund Firsching

**Affiliations:** 1 Department of Neurosurgery, Faculty of Medicine, Otto von Guericke University Magdeburg, Magdeburg, Germany; 2 Institute of Biometry and Medical Informatics, Faculty of Medicine, Otto von Guericke University Magdeburg, Magdeburg, Germany; 3 Department of Neurology, Faculty of Medicine, Otto von Guericke University Magdeburg, Magdeburg, Germany; 4 Department of Neurology, Max Planck Institute for Human Cognitive and Brain Sciences, Leipzig, Germany; University of Jaén, Spain

## Abstract

Chronic pain is one of the most common health complaints in industrial nations. For example, chronic low back pain (cLBP) disables millions of people across the world and generates a tremendous economic burden. While previous studies provided evidence of widespread functional as well as structural brain alterations in chronic pain, little is known about cortical changes in patients suffering from lumbar disc herniation. We investigated morphometric alterations of the gray and white matter of the brain in patients suffering from LDH. The volumes of the gray and white matter of 12 LDH patients were determined in a prospective study and compared to the volumes of healthy controls to distinguish local differences. High-resolution MRI brain images of all participants were performed using a 3 Tesla MRI scanner. Voxel-based morphometry was used to investigate local differences in gray and white matter volume between patients suffering from LDH and healthy controls. LDH patients showed significantly reduced gray matter volume in the right anterolateral prefrontal cortex, the right temporal lobe, the left premotor cortex, the right caudate nucleus, and the right cerebellum as compared to healthy controls. Increased gray matter volume, however, was found in the right dorsal anterior cingulate cortex, the left precuneal cortex, the left fusiform gyrus, and the right brainstem. Additionally, small subcortical decreases of the white matter were found adjacent to the left prefrontal cortex, the right premotor cortex and in the anterior limb of the left internal capsule. We conclude that the lumbar disk herniation can lead to specific local alterations of the gray and white matter in the human brain. The investigation of LDH-induced brain alterations could provide further insight into the underlying nature of the chronification processes and could possibly identify prognostic factors that may improve the conservative as well as the operative treatment of the LDH.

## Introduction

Chronic low back pain (cLBP) disables millions of people across the world and generates a tremendous economic burden. Recent studies estimated the direct and indirect costs of back pain up to $624.8 billion [1], [2]. Less than 5% of cLBP patients suffer from lumbar disc herniation (LDH). Nevertheless, more than 30% of annual costs for the medical care of cLBP are spent on conservative as well as invasive treatment strategies in this specific patient population [3]. Although previous findings showed that herniated intervertebral discs occur in asymptomatic patients [4], [5] several authors defined selection criteria for surgical treatment based on classification of the magnetic resonance imaging (MRI) findings of the spine [6]–[10]. Previous studies compared the efficiency of non-surgical management and surgical treatment of LDH [11]–[15]. Thus, surgical treatment tends to improve symptoms like pain and motor weakness more efficiently than conservative management. The differences, however, were small and not always statistically significant for the outcome and surgery did not improve the return-to-work rate as compared to non-surgical treatment. Additionally, in a very recent study El Barzouhi et al. [16] revealed that the MRI assessment of disk herniation one year after the treatment did not distinguish between patients with a favorable outcome and those with an unfavorable outcome. Unfortunately, until now no factors were identified that can predict which patients are likely to improve by a non-surgical management and which should better be treated with surgery [17]–[19].

The adult brain has a remarkable capacity for morphological alterations following learning and adaption processes to a changed environment [20]–[22]. An increasingly large body of evidence indicates that functional and structural brain adaptations play a crucial role in mediating acute and chronic pain disorders. In recent studies, acute pain has been associated with structural as well as functional brain alterations predominantly in the somatosensory system, particularly the thalamus [23], [24]. Chronic pain, however, appears to be more complex. Previous studies using functional and structural brain imaging showed that chronic regional pain is accompanied by dysfunctions of central inhibitory and modulatory systems [23]–[30]. Ung et al. [31] revealed that these changes are specific and therefore can be classified for diagnostic reasons. Cortical changes of the brain resulting from back pain have the potential to enhance our understanding of the neuropathology of cLBP and therefore optimize conservative as well as surgical strategies. To date, no study has directly investigated possible morphometric changes of the brain associated with chronic pain due to lumbar disc herniation. In the present study we investigated gray and white matter alterations in the brain in patients with chronic back pain resulting from LDH.

## Patients and Methods

The study was approved by the Local Ethics Committee of the Medical Faculty of the University of Magdeburg in compliance with national legislation and the Code of Ethical Principles for Medical Research Involving Human Subjects of the World Medical Association (Declaration of Helsinki).

### Subjects

The study comprised two groups: 12 patients (mean age 43.9±12.9 years) with chronic low back pain were enrolled in the study. The individual duration of the back pain exceeded at least 3 month. All patients were diagnosed with isolated lumbar disc herniation either at the level L4–5 or L5-S1 using spinal MRI. The second group consisted of 12 age- and gender-matched subjects and served as control group. No member of the control group had suffered from any back pain or neurological disorder. All participants gave their informed written consent. All subjects were neurologically examined prior to the MR imaging. The current mean level of back pain was quantified with the visual analogue scale (VAS).

### MR Imaging

MR imaging was performed on a 3 Tesla Siemens Magnetom Trio scanner (Erlangen, Germany) using an 8-channel phased array head coil. A high-resolution anatomical dataset of the entire brain was acquired using a T1-weighted magnetization prepared rapid gradient echo (MPRAGE) sequence (field of view = 256 mm, matrix size = 256×256, slices = 192, slice thickness = 1 mm, repetition time = 2500 ms, flip angle = 7%) resulting in an isotropic resolution of 1 mm^3^.

### MRI Data Preprocessing

MRI data was analyzed using the voxel-based morphometry toolbox (VBM8 [32]) implemented in SPM8 running under Matlab. 5 of 12 patients had left sciatica due to left mediolateral disc herniation. In order to achieve a comparable group, the images of these patients were flipped. In brief, the T1-weighted MR images of all subjects were bias corrected, segmented, and finally registered to the standardized Montreal Neurological Institute (MNI) space using the segmentation approach by Ashburner and Friston [33]. Gray matter (GM) and white matter (WM) segments were scaled using the Jacobian determinants of the deformation to account for distortions during linear and non-linear transformation [33]. Finally, the modulated GM and WM densities were smoothed using an 8 mm FWHM (full width at half maximum) Gaussian kernel. For subsequent statistical analysis, we excluded all voxels with a GM value below 0.2 (with a maximum value of 1) to avoid possible partial volume effects near the border between GM and WM.

### MRI Data Analyses

Differences of the GM and WM between both groups were estimated using a univariate voxel-based two-sample-t-test. The GM and WM volumes were compared as absolute units. In order to allocate the cytoarchitectonic reference the anatomical SPM toolbox by Eickhoff et al. [34] was used. All data and images are displayed in neurological convention.

## Results

The control group reported a mean pain intensity of 0 and the neurological examination revealed no neurological deficits. LDH patients reported a mean pain intensity level of 7.2±0.9 ranging between 6 and 8. All patients suffered from low back pain and sciatica that radiated at least into one leg in a classic dermatomal distribution. In all patients, the pain was accompanied by numbness and tingling for at least 3 month. However, the clinical examination revealed no muscle weaknesses in any of the patients. The MRI examination of the spine discovered lumbar disc herniation at the level L4–5 in 5 patients and at the level L5-S1 in 7 patients.

### Analyses of Gray and White Matter Changes

**Figure 1:**
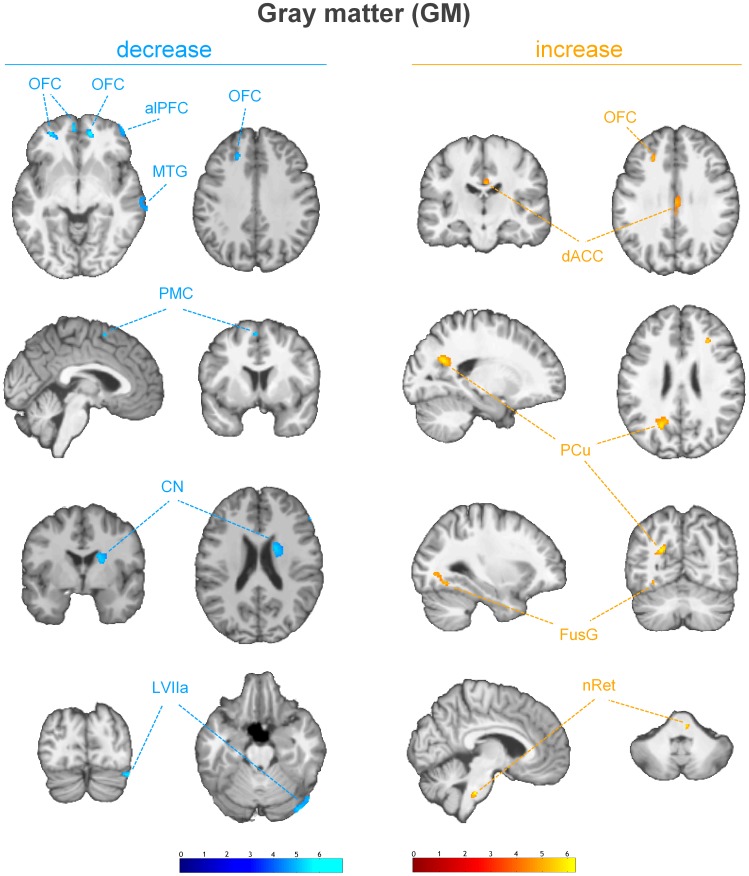
Decrease (blue) and increase (red) of gray matter volume in LDH patients relative to healthy controls. (*p*<0.0001, uncorrected for multiple comparison, cluster threshold of 15 voxel) The group analysis showed decreased gray matter volumes in the right anterolateral prefrontal cortex (alPFC), the right middle temporal gyrus (MTG), the left premotor cortex (PMC), the right caudate nucleus (CN), and the lobule VIIa (Crus I) of the right cerebellum (LVIIa). Increases were found in the right dorsal anterior cingulate cortex (dACC), the left lateral precuneal region (PCu), the left fusiform gyrus (FusG), and the nucleus reticularis of the right brainstem at the level of the basal pons (nRet). Bilateral increases as well as decreases are shown in the orbitofrontal cortex (OFC). [Images are presented in neurological convention].

**Figure 2:**
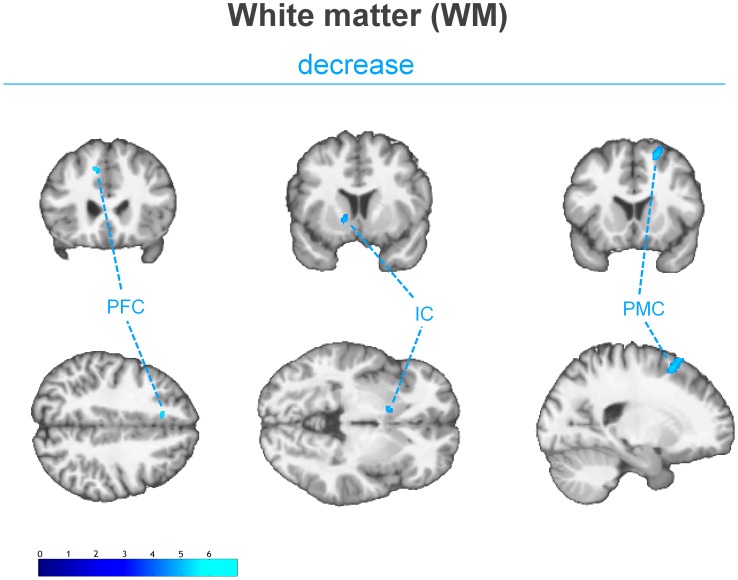
Decrease of the white matter volume in LDH patients relative healthy controls. (*p*<0.0001, uncorrected for multiple comparison, cluster threshold of 15 voxel) The group analysis showed decreased subcortical white matter volumes adjacent to the left prefrontal cortex (PFC), the right premotor cortex (PMC) and the anterior limb of the left internal capsule (IC). [Images are presented in neurological convention].

The examination of the T1-weighted MRI data of all subjects revealed no pathological brain alterations. Figure 1 and Table 1 show the results of the group-level analysis of gray matter volume changes. Compared to healthy controls, LDH patients showed significantly reduced gray matter volume in the right anterolateral prefrontal cortex (alPFC), the right temporal lobe, the left premotor cortex (PMC), the right caudate nucleus (CN), and the right cerebellum. Increased gray matter volume, however, was found in the right dorsal anterior cingulate cortex (dACC), the left precuneal cortex (PCu), the left fusiform gyrus (FusG), and the right brainstem. Additionally, bilaterally decreased as well as increased gray matter volume was found in the orbitofrontal cortex (OFC). Figure 2 shows the statistically significant changes of the white matter volume. Small subcortical decreases were found in the frontal lobes and in the anterior limb of the left internal capsule.

**Table 1:** Significant gray and white matter changes in LDH patients relative to healthy controls.

Region	WM/GM	change	MNI coordinates in mm			Z-score of peak-change
			x	y	z	
caudate nucleus	GM	decrease	22	20	7	4.51
cerebellum	GM	decrease	42	−84	−24	4.42
middle temporal gyrus	GM	decrease	66	−21	−12	4.60
orbitofrontal cortex	GM	decrease	−21	45	−9	3.98
	GM	decrease	−4	57	−5	4.44
	GM	decrease	14	44	−12	4.93
(subcortical)	WM	decrease	21	15	55	4.49
	GM	increase	30	26	27	4.41
anterolateral prefrontal cortex (alPFC)	GM	decrease	51	52	−6	4.04
premotor cortex	GM	decrease	−2	5	58	3.85
(subcortical)	WM	decrease	−10	23	40	5.10
dorsal anterior cingulate cortex (dACC)	GM	increase	6	−9	33	4.40
precuneus	GM	increase	−21	−64	24	4.60
fusiform gyrus	GM	increase	−28	−78	−2	4.12
Brainstem	GM	increase	6	−31	−41	4.63
internal capsule	WM	decrease	−12	8	−5	4.38

## Discussion

Using voxel-based morphometry [32] our study showed significant local differences in gray and white matter volume between patients suffering from chronic low back pain (cLBP) and sciatica due to lumbar disc herniation (LDH) as compared to healthy controls. The findings can be divided into three main groups: (1) areas with decreased gray matter in LDH patients compared to healthy control group such as the right caudate nucleus and parts of the right cerebellum; (2) areas showing increased gray matter volume mainly involving the right dorsal anterior cingulate cortex (dACC), the left lateral precuneal region and the brainstem, and (3) regions that show decreased white matter volume such as in the anterior limb of the left internal capsule. Until recently, it has been hypothesized that chronic pain is associated with abnormal nociceptive and antinociceptive function but preserved normal brain structure [35]. The present findings support the contemporary assumption that chronic pain may lead to altered morphology and/or cytoarchitecture in brain regions that are crucial to pain perception and processing [29], [35]–[41]. The physiology of pain and pathophysiology of chronic pain is increasingly understood not only as an altered functional state, but also as a consequence of plasticity in the central nervous system [29].

The present findings are consistent with most morphometric studies investigating structural brain plasticity in patients suffering from chronic pain [38]. As displayed in Figure 1 LDH patients showed an increased gray matter volume in the dorsal anterior cingulate cortex (dACC). These findings are highly consistent with recent studies. Teutsch et al. [35] observed a significant gray matter increase in the medial cingulate cortex resulting from repetitive painful stimulation. Bingel et al. [42] showed increased BOLD responses to repetitive nociceptive stimuli in the anterior cingulate cortex using functional magnetic resonance imaging (fMRI). Hence, the anterior cingulate cortex has been assumed to play a possibly predominant role in endogen pain control and habituation processing to chronic pain. Additionally, it is an intriguing observation that chronic opioid exposure leads to a dose-dependent gray matter increase in the cingulate cortex [43]. Interestingly, only one of the twelve patients used opioids for the treatment of chronic low back pain. The other patients used non-steroidal anti-inflammatory drugs (NSAID) or renounced the frequent use of painkillers. A significant increase of gray matter volume was also found in the precuneus. This region is known to be involved in a wide spectrum of highly integrated tasks, including episodic memory retrieval and self-processing operations [44]. Several studies showed structural as well as functional alterations in this region [42], [45], [46]. Based on a Pavlovian conditioning study Kattoor et al. [47] proposed a central fear network including the anterior cingulate cortex (ACC) and the precuneus during associative learning. These regions are known to be crucially involved in the coordination of affective reactions to painful stimuli by encoding emotional and motivational demands [48], which are therefore thought to specifically mediate the fear avoidance aspect of pain processing [49]. The brainstem is another region showing increased gray matter compared to the healthy controls. Several studies revealed pain-specific connectivity between the brainstem and cortical networks that are involved in pain processing using fMRI [50]–[52]. The findings are in line with the observations of several groups [53], [54] who found alterations of gray matter in the brainstem of patients suffering from chronic back pain indicating that prolonged pain perception is associated with cortical and subcortical reorganization. These structural alterations may play an important role in the process of the chronification of pain [39]. In contrast to these authors, we found an increase of gray matter in the brainstem. The patients of the present study suffered from neuropathic pain resulting from LDH. While Kim et al. [53] observed the gray matter decrease in patients with migraine the peripheral cause of cLBP in LDH patients might be an explanation for pain-specific changes of gray matter in the brainstem. In contrast to the brainstem a decreased gray matter volume of the right anterolateral prefrontal cortex (alPFC) was found in LDH patients. This region is believed to mediate analgesic effects of expected and perceived control over pain. Wiech et al. [55] described a neural basis for the analgesic effects of perceived pain control. The authors suggest that a decreased activation in the right alPFC may explain the maladaptive effect of unsuccessful coping during uncontrollable pain. Another decrease of gray matter was found in the right caudate nucleus. The caudate nucleus as part of the striatum is located in the basal ganglia (BG). A large body of evidence suggests that the basal ganglia are uniquely involved in thalamo-cortico-BG loops to integrate motor, emotional, autonomic and cognitive aspects of chronic pain [56]. Specific pain-induced activations in the caudate nucleus have been suggested to be a crucial part of the pain modulatory system of the brain [57], [58]. The authors indicate that these activations may reduce the affective components of the pain [56]. Thus, the detected decreased volume of gray matter could lead to a decreased activation in the right striatum. Subcortical structural changes in these regions may be associated with uncontrollable chronic pain. In contrast to some previous studies we found a decreased gray matter volume in the striatum. Increases in gray matter in the basal ganglia have been reported in patients suffering from fibromyalgia [40] and chronic vulval pain [59]. This lack of consistency is difficult to explain, but may be due to different neuropathological mechanisms of the processes ultimately leading to pain chronification. Further gray matter decreases were found in lobule VIIa of the right cerebellum. Recent studies likewise found decreased gray matter volume in patients with chronic tension type headache, migraine and trigeminal neuralgia [60]–[62]. Interestingly, the decreased volume correlated positively with the duration of the headache. Some authors proposed a relationship between cerebellar abnormalities and the metabolic and functional disturbances in migraine [63], [64]. Functional MRI evidence for pain-related pattern in the cerebellum has been reported. Gracely et al. [65] showed that coping strategies like catastrophizing lead to altered activations in the cerebellum. A direct relationship between cLBP and structural changes in the cerebellum has not been established yet. Diffuse decreases as well as increases of the gray and white matters were found in the orbitofrontal cortex. Our results are in line with recent studies [29], [36], [40], [46] indicating a pain-modulating role in the antinociceptive system [66], [67]. The analysis of the white matter revealed a decrease in the anterior limb of the left internal capsule. This region contains bundles of fibers that connect the frontal lobe with regions such as the caudate where significant decreases of the gray matter volume were found.

Interestingly, no GM and WM changes were found in the primary somatosensory cortex. While short lasting pain leads to substantial increase of the gray matter in the somatosensory cortex [35] no changes were found in patients suffering from chronic pain. As things are now, it is unknown why some people develop chronic pain syndromes. It seems that chronic pain patients lost the ability to habituate to pain [68], [69]. Some authors explain the lack of GM increase in the somatosensory cortex with cognitive maladaptation to acute pain. They speculate that no significant noxious input is present any more. Probably, the brain mostly drives the experience of a constant pain [35]. This idea is consistent with the assumption that the fear of pain is more disabling than the pain itself [70]. The contemporary fear-avoidance-model was introduced to describe how pain-related fear leads to pain disability, affective distress and physical disuse as result of persistent avoidance behaviors [71].

The current study has some limitations that need to be addressed. The nature of cross-sectional studies allows only limited conclusions on the dynamic pattern of the observed cerebral changes. Despite the significant structural differences between the LDH patients and the healthy control group the VBM analysis cannot account for the underlying cytoarchitectonic reasons. Thus, a variable size of cells, increased or decreased synaptogenesis or an altered number of astro- and microglia might account for the GM and WM changes found [40]. Additionally, the results and therefore the observed regions are limited by a relatively small statistical power. Some of the regions that were found could merely related to high levels of noise. Nevertheless, the presented exploratory approach and results justify large-scale studies. Thus, further multimodal imaging studies should be conducted to provide further insight into the underlying nature of these pain-related morphometric brain alterations and could possibly identify prognostic factors that improve the conservative as well as the operative treatment of the LDH.

## References

[1] Wenig CM, Schmidt CO, Kohlmann T, Schweikert B (2009) Costs of back pain in Germany. Eur J Pain 13: 280–286. 18524652 10.1016/j.ejpain.2008.04.005

[2] Dagenais S, Caro J, Haldeman S (2008) A systematic review of low back pain cost of illness studies in the United States and internationally. Spine J 8: 8–20. 18164449 10.1016/j.spinee.2007.10.005

[3] Shvartzman L, Weingarten E, Sherry H, Levin S, Persaud A (1992) Cost-effectiveness analysis of extended conservative therapy versus surgical intervention in the management of herniated lumbar intervertebral disc. Spine (Phila Pa 1976) 17: 176–182. 1532460 10.1097/00007632-199202000-00010

[4] Boden SD, Davis DO, Dina TS, Patronas NJ, Wiesel SW (1990) Abnormal magnetic-resonance scans of the lumbar spine in asymptomatic subjects. A prospective investigation. J Bone Joint Surg Am 72: 403–408. 2312537

[5] Jensen MC, Brant-Zawadzki MN, Obuchowski N, Modic MT, Malkasian D, et al (1994) Magnetic resonance imaging of the lumbar spine in people without back pain. N Engl J Med 331: 69–73. 8208267 10.1056/NEJM199407143310201

[6] Carragee EJ, Kim DH (1997) A prospective analysis of magnetic resonance imaging findings in patients with sciatica and lumbar disc herniation. Correlation of outcomes with disc fragment and canal morphology. Spine (Phila Pa 1976) 22: 1650–1660. 9253102 10.1097/00007632-199707150-00025

[7] Karppinen J, Ohinmaa A, Malmivaara A, Kurunlahti M, Kyllonen E, et al (2001) Cost effectiveness of periradicular infiltration for sciatica: subgroup analysis of a randomized controlled trial. Spine (Phila Pa 1976) 26: 2587–2595. 11725240 10.1097/00007632-200112010-00013

[8] Kortelainen P, Puranen J, Koivisto E, Lahde S (1985) Symptoms and signs of sciatica and their relation to the localization of the lumbar disc herniation. Spine (Phila Pa 1976) 10: 88–92. 3983706 10.1097/00007632-198501000-00014

[9] Mysliwiec LW, Cholewicki J, Winkelpleck MD, Eis GP (2010) MSU classification for herniated lumbar discs on MRI: toward developing objective criteria for surgical selection. Eur Spine J 19: 1087–1093. 20084410 10.1007/s00586-009-1274-4 PMC2900017

[10] Ohnmeiss DD, Vanharanta H, Ekholm J (1997) Degree of disc disruption and lower extremity pain. Spine (Phila Pa 1976) 22: 1600–1605. 9253095 10.1097/00007632-199707150-00015

[11] Atlas SJ, Keller RB, Chang Y, Deyo RA, Singer DE (2001) Surgical and nonsurgical management of sciatica secondary to a lumbar disc herniation: five-year outcomes from the Maine Lumbar Spine Study. Spine (Phila Pa 1976) 26: 1179–1187. 11413434 10.1097/00007632-200105150-00017

[12] Atlas SJ, Keller RB, Wu YA, Deyo RA, Singer DE (2005) Long-term outcomes of surgical and nonsurgical management of lumbar spinal stenosis: 8 to 10 year results from the maine lumbar spine study. Spine (Phila Pa 1976) 30: 936–943. 15834339 10.1097/01.brs.0000158953.57966.c0

[13] Peul WC, van Houwelingen HC, van den Hout WB, Brand R, Eekhof JAH, et al (2007) Surgery versus prolonged conservative treatment for sciatica. N Engl J Med 356: 2245–2256. 17538084 10.1056/NEJMoa064039

[14] Weinstein JN, Lurie JD, Tosteson TD, Tosteson ANA, Blood EA, et al (2008) Surgical versus nonoperative treatment for lumbar disc herniation: four-year results for the Spine Patient Outcomes Research Trial (SPORT). Spine (Phila Pa 1976) 33: 2789–2800. 19018250 10.1097/BRS.0b013e31818ed8f4 PMC2756172

[15] Weinstein JN, Tosteson TD, Lurie JD, Tosteson AN, Hanscom B, et al (2006) Surgical vs nonoperative treatment for lumbar disk herniation: the Spine Patient Outcomes Research Trial (SPORT): a randomized trial. JAMA 296: 2441–2450. 17119140 10.1001/jama.296.20.2441 PMC2553805

[16] el Barzouhi A, Vleggeert-Lankamp CL, Lycklama a Nijeholt GJ, Van der Kallen BF, van den Hout WB, et al (2013) Magnetic resonance imaging in follow-up assessment of sciatica. N Engl J Med 368: 999–1007. 23484826 10.1056/NEJMoa1209250

[17] Moschetti WP AM, Abdu WA (2009) Treatment of Lumbar Disc Herniation: An Evidence-Based Review. Seminars in Spine Surgery 21: 223–229.

[18] Sonntag VK (2010) Treatment of the herniated lumbar disc: persistent problem. World Neurosurg 74: 574–575. 21492621 10.1016/j.wneu.2010.08.006

[19] Sonntag VK (2012) Lumbar and cervical disc herniations: a common problem, many solutions. World Neurosurg 77: 71–72. 22405388 10.1016/j.wneu.2011.06.007

[20] Markham JA, Greenough WT (2004) Experience-driven brain plasticity: beyond the synapse. Neuron glia biology 1: 351–363. 16921405 10.1017/s1740925x05000219 PMC1550735

[21] Adkins DL, Boychuk J, Remple MS, Kleim JA (2006) Motor training induces experience-specific patterns of plasticity across motor cortex and spinal cord. J Appl Physiol 101: 1776–1782. 16959909 10.1152/japplphysiol.00515.2006

[22] Draganski B, May A (2008) Training-induced structural changes in the adult human brain. Behavioural brain research 192: 137–142. 18378330 10.1016/j.bbr.2008.02.015

[23] Apkarian AV, Bushnell MC, Treede R-D, Zubieta J-K (2005) Human brain mechanisms of pain perception and regulation in health and disease. Eur J Pain 9: 463–484. 15979027 10.1016/j.ejpain.2004.11.001

[24] deCharms RC, Maeda F, Glover GH, Ludlow D, Pauly JM, et al (2005) Control over brain activation and pain learned by using real-time functional MRI. Proc Natl Acad Sci U S A 102: 18626–18631. 16352728 10.1073/pnas.0505210102 PMC1311906

[25] Borsook D, Sava S, Becerra L (2010) The pain imaging revolution: advancing pain into the 21st century. Neuroscientist 16: 171–185. 20400714 10.1177/1073858409349902 PMC3370428

[26] Giesecke T, Gracely RH, Williams DA, Geisser ME, Petzke FW, et al (2005) The relationship between depression, clinical pain, and experimental pain in a chronic pain cohort. Arthritis Rheum 52: 1577–1584. 15880832 10.1002/art.21008

[27] Gracely RH, Petzke F, Wolf JM, Clauw DJ (2002) Functional magnetic resonance imaging evidence of augmented pain processing in fibromyalgia. Arthritis Rheum 46: 1333–1343. 12115241 10.1002/art.10225

[28] Ingvar M (1999) Pain and functional imaging. Philos Trans R Soc Lond B Biol Sci 354: 1347–1358. 10466155 10.1098/rstb.1999.0483 PMC1692633

[29] May A (2008) Chronic pain may change the structure of the brain. Pain 137: 7–15. 18410991 10.1016/j.pain.2008.02.034

[30] Wood PB (2010) Variations in brain gray matter associated with chronic pain. Curr Rheumatol Rep 12: 462–469. 20857244 10.1007/s11926-010-0129-7

[31] Ung H, Brown JE, Johnson KA, Younger J, Hush J, et al.. (2012) Multivariate Classification of Structural MRI Data Detects Chronic Low Back Pain. Cereb Cortex. 10.1093/cercor/bhs378 PMC3948494 23246778

[32] Ashburner J, Friston KJ (2000) Voxel-based morphometry–the methods. Neuroimage 11: 805–821. 10860804 10.1006/nimg.2000.0582

[33] Ashburner J, Friston KJ (2005) Unified segmentation. Neuroimage 26: 839–851. 15955494 10.1016/j.neuroimage.2005.02.018

[34] Eickhoff SB, Stephan KE, Mohlberg H, Grefkes C, Fink GR, et al (2005) A new SPM toolbox for combining probabilistic cytoarchitectonic maps and functional imaging data. Neuroimage 25: 1325–1335. 15850749 10.1016/j.neuroimage.2004.12.034

[35] Teutsch S, Herken W, Bingel U, Schoell E, May A (2008) Changes in brain gray matter due to repetitive painful stimulation. Neuroimage 42: 845–849. 18582579 10.1016/j.neuroimage.2008.05.044

[36] Apkarian AV, Sosa Y, Sonty S, Levy RM, Harden RN, et al (2004) Chronic back pain is associated with decreased prefrontal and thalamic gray matter density. J Neurosci 24: 10410–10415. 15548656 10.1523/JNEUROSCI.2541-04.2004 PMC6730296

[37] Kuchinad A, Schweinhardt P, Seminowicz DA, Wood PB, Chizh BA, et al (2007) Accelerated brain gray matter loss in fibromyalgia patients: premature aging of the brain? J Neurosci 27: 4004–4007. 17428976 10.1523/JNEUROSCI.0098-07.2007 PMC6672521

[38] May A (2011) Structural brain imaging: a window into chronic pain. Neuroscientist 17: 209–220. 21489967 10.1177/1073858410396220

[39] Schmidt-Wilcke T, Leinisch E, Ganssbauer S, Draganski B, Bogdahn U, et al (2006) Affective components and intensity of pain correlate with structural differences in gray matter in chronic back pain patients. Pain 125: 89–97. 16750298 10.1016/j.pain.2006.05.004

[40] Schmidt-Wilcke T, Luerding R, Weigand T, Jurgens T, Schuierer G, et al (2007) Striatal grey matter increase in patients suffering from fibromyalgia–a voxel-based morphometry study. Pain 132 Suppl 1109–116. 10.1016/j.pain.2007.05.010 17587497

[41] Wand BM, Parkitny L, O’Connell NE, Luomajoki H, McAuley JH, et al (2011) Cortical changes in chronic low back pain: current state of the art and implications for clinical practice. Man Ther 16: 15–20. 20655796 10.1016/j.math.2010.06.008

[42] Bingel U, Schoell E, Herken W, Buchel C, May A (2007) Habituation to painful stimulation involves the antinociceptive system. Pain 131: 21–30. 17258858 10.1016/j.pain.2006.12.005

[43] Younger JW, Chu LF, D’Arcy NT, Trott KE, Jastrzab LE, et al (2011) Prescription opioid analgesics rapidly change the human brain. Pain 152: 1803–1810. 21531077 10.1016/j.pain.2011.03.028 PMC3138838

[44] Cavanna AE, Trimble MR (2006) The precuneus: a review of its functional anatomy and behavioural correlates. Brain 129: 564–583. 16399806 10.1093/brain/awl004

[45] Duerden EG, Albanese M-C (2013) Localization of pain-related brain activation: a meta-analysis of neuroimaging data. Hum Brain Mapp 34: 109–149. 22131304 10.1002/hbm.21416 PMC6869965

[46] Rodriguez-Raecke R, Niemeier A, Ihle K, Ruether W, May A (2013) Structural brain changes in chronic pain reflect probably neither damage nor atrophy. PLoS One 8: e54475. 23405082 10.1371/journal.pone.0054475 PMC3566164

[47] Kattoor J, Gizewski ER, Kotsis V, Benson S, Gramsch C, et al (2013) Fear conditioning in an abdominal pain model: neural responses during associative learning and extinction in healthy subjects. PLoS One 8: e51149. 23468832 10.1371/journal.pone.0051149 PMC3582635

[48] Shackman AJ, Salomons TV, Slagter HA, Fox AS, Winter JJ, et al (2011) The integration of negative affect, pain and cognitive control in the cingulate cortex. Nat Rev Neurosci 12: 154–167. 21331082 10.1038/nrn2994 PMC3044650

[49] Vogt BA (2005) Pain and emotion interactions in subregions of the cingulate gyrus. Nat Rev Neurosci 6: 533–544. 15995724 10.1038/nrn1704 PMC2659949

[50] Dunckley P, Wise RG, Fairhurst M, Hobden P, Aziz Q, et al (2005) A comparison of visceral and somatic pain processing in the human brainstem using functional magnetic resonance imaging. J Neurosci 25: 7333–7341. 16093383 10.1523/JNEUROSCI.1100-05.2005 PMC6725297

[51] Fairhurst M, Wiech K, Dunckley P, Tracey I (2007) Anticipatory brainstem activity predicts neural processing of pain in humans. Pain 128: 101–110. 17070996 10.1016/j.pain.2006.09.001

[52] Petrovic P, Petersson KM, Hansson P, Ingvar M (2004) Brainstem involvement in the initial response to pain. Neuroimage 22: 995–991005. 15193631 10.1016/j.neuroimage.2004.01.046

[53] Kim JH, Suh SI, Seol HY, Oh K, Seo WK, et al (2008) Regional grey matter changes in patients with migraine: a voxel-based morphometry study. Cephalalgia 28: 598–604. 18422725 10.1111/j.1468-2982.2008.01550.x

[54] Rodriguez-Raecke R, Niemeier A, Ihle K, Ruether W, May A (2009) Brain gray matter decrease in chronic pain is the consequence and not the cause of pain. J Neurosci 29: 13746–13750. 19889986 10.1523/JNEUROSCI.3687-09.2009 PMC6666725

[55] Wiech K, Kalisch R, Weiskopf N, Pleger B, Stephan KE, et al (2006) Anterolateral prefrontal cortex mediates the analgesic effect of expected and perceived control over pain. J Neurosci 26: 11501–11509. 17079679 10.1523/JNEUROSCI.2568-06.2006 PMC2635565

[56] Borsook D, Upadhyay J, Chudler EH, Becerra L (2010) A key role of the basal ganglia in pain and analgesia–insights gained through human functional imaging. Mol Pain 6: 27–27. 20465845 10.1186/1744-8069-6-27 PMC2883978

[57] Freund W, Klug R, Weber F, Stuber G, Schmitz B, et al (2009) Perception and suppression of thermally induced pain: a fMRI study. Somatosens Mot Res 26: 1–10. 19283551 10.1080/08990220902738243

[58] Freund W, Stuber G, Wunderlich AP, Schmitz B (2007) Cortical correlates of perception and suppression of electrically induced pain. Somatosens Mot Res 24: 203–212. 18097993 10.1080/08990220701723636

[59] Schweinhardt P, Kuchinad A, Pukall CF, Bushnell MC (2008) Increased gray matter density in young women with chronic vulvar pain. Pain 140: 411–419. 18930351 10.1016/j.pain.2008.09.014

[60] Schmidt-Wilcke T, Leinisch E, Straube A, Kampfe N, Draganski B, et al (2005) Gray matter decrease in patients with chronic tension type headache. Neurology 65: 1483–1486. 16275843 10.1212/01.wnl.0000183067.94400.80

[61] Jin C, Yuan K, Zhao L, Zhao L, Yu D, et al (2013) Structural and functional abnormalities in migraine patients without aura. NMR Biomed 26: 58–64. 22674568 10.1002/nbm.2819

[62] Obermann M, Rodriguez-Raecke R, Naegel S, Holle D, Mueller D, et al (2013) Gray matter volume reduction reflects chronic pain in trigeminal neuralgia. Neuroimage 74: 352–358. 23485849 10.1016/j.neuroimage.2013.02.029

[63] Sandor PS, Mascia A, Seidel L, de Pasqua V, Schoenen J (2001) Subclinical cerebellar impairment in the common types of migraine: a three-dimensional analysis of reaching movements. Ann Neurol 49: 668–672. 11357959

[64] Dichgans M, Herzog J, Freilinger T, Wilke M, Auer DP (2005) 1H-MRS alterations in the cerebellum of patients with familial hemiplegic migraine type 1. Neurology 64: 608–613. 15728280 10.1212/01.WNL.0000151855.98318.50

[65] Gracely RH, Geisser ME, Giesecke T, Grant MAB, Petzke F, et al (2004) Pain catastrophizing and neural responses to pain among persons with fibromyalgia. Brain 127: 835–843. 14960499 10.1093/brain/awh098

[66] Petrovic P, Ingvar M (2002) Imaging cognitive modulation of pain processing. Pain 95: 1–5. 11790461 10.1016/s0304-3959(01)00467-5

[67] Lorenz J, Minoshima S, Casey KL (2003) Keeping pain out of mind: the role of the dorsolateral prefrontal cortex in pain modulation. Brain 126: 1079–1091. 12690048 10.1093/brain/awg102

[68] Peters ML, Schmidt AJ, Van den Hout MA (1989) Chronic low back pain and the reaction to repeated acute pain stimulation. Pain 39: 69–76. 2530488 10.1016/0304-3959(89)90176-0

[69] Flor H, Diers M, Birbaumer N (2004) Peripheral and electrocortical responses to painful and non-painful stimulation in chronic pain patients, tension headache patients and healthy controls. Neurosci Lett 361: 147–150. 15135915 10.1016/j.neulet.2003.12.064

[70] Crombez G, Vlaeyen JW, Heuts PH, Lysens R (1999) Pain-related fear is more disabling than pain itself: evidence on the role of pain-related fear in chronic back pain disability. Pain 80: 329–339. 10204746 10.1016/s0304-3959(98)00229-2

[71] Vlaeyen JW, Linton SJ (2012) Fear-avoidance model of chronic musculoskeletal pain: 12 years on. Pain 153: 1144–1147. 22321917 10.1016/j.pain.2011.12.009

